# Screening of Insertion Sites and Tags on EV-A71 VP1 Protein for Recombinant Virus Construction

**DOI:** 10.3390/v17010128

**Published:** 2025-01-17

**Authors:** Miaomiao Kang, Xiangyi Li, Xiaohong Li, Rui Yu, Shuo Zhang, Jingjing Yan, Xiaoyan Zhang, Jianqing Xu, Buyong Ma, Shuye Zhang

**Affiliations:** 1 Clinical Center for Biotherapy, Zhongshan Hospital, Fudan University, Shanghai 200433, China; kangmiaomiao2024@163.com (M.K.); 23111210007@m.fudan.edu.cn (X.L.); 21211300001@m.fudan.edu.cn (S.Z.); xujianqing@fudan.edu.cn (J.X.); 2 Engineering Research Center of Cell & Therapeutic Antibody (MOE), School of Pharmacy, Shanghai Jiao Tong University, Shanghai 200240, China; waterrr-lxy@sjtu.edu.cn; 3 Patronus Biotech Co., Ltd., Guangzhou 510715, China; bioyurui@163.com; 4 Shanghai Public Health Clinical Center, Fudan University, Shanghai 201508, China; yanjing872006@126.com (J.Y.); zhangxiaoyan@fudan.edu.cn (X.Z.)

**Keywords:** Enterovirus A71, capsids, tags

## Abstract

This study aimed to create a new recombinant virus by modifying the EV-A71 capsid protein, serving as a useful tool and model for studying human Enteroviruses. We developed a new screening method using EV-A71 pseudovirus particles to systematically identify suitable insertion sites and tag types in the VP1 capsid protein. The pseudovirus’s infectivity and replication can be assessed by measuring postinfection luciferase signals. We reported that the site after the 100th amino acid within the VP1 BC loop of EV-A71 is particularly permissive for the insertion of various tags. Notably, the introduction of S and V5 tags at this position had minimal effect on the fitness of the tagged pseudovirus. Furthermore, recombinant infectious EV-A71 strains tagged with S and V5 epitopes were successfully rescued, and the stability of these tags was verified. Computational analysis suggested that viable insertions should be compatible with capsid assembly and receptor binding, whereas non-viable insertions could potentially disrupt the capsid’s binding with heparan sulfate. We expect the tagged recombinant EV-A71 to be a useful tool for studying the various stages of the enterovirus life cycle and for virus purification, immunoprecipitation, and research in immunology and vaccine development. Furthermore, this study serves as a proof of principle and may help develop similar tags in enteroviruses, for which there are fewer available tools.

## 1. Introduction

Human Enterovirus, a member of the *Picornaviridae* family, is classified into human *Enterovirus species A*, *B*, *C*, and *D*, as well as *Rhinovirus species A*, *B*, and *C* [1]. Enteroviruses are a class of non-enveloped RNA viruses characterized by a naked capsid and a genome roughly 7.5 kb in length [2]. This virus group includes several significant human pathogens, such as Enterovirus A71 (EV-A71), Coxsackievirus B3 (CVB-3), Poliovirus (PV), and Enterovirus D68 (EV-D68), among others. These viruses can cause a range of symptoms and diseases [3,4,5,6]. Currently, there are no specific treatments for enterovirus infections. Although the inactivated vaccine for EV-A71 has been approved and applied in China, studies have shown that the EV-A71 vaccine cannot induce cross-protection against infections from other serotypes [7,8].

The preparation of recombinant viruses carrying foreign epitopes through viral reverse genetics techniques provides important tools for studying the functions of viral proteins. Most previous studies have reported the incorporation of tags into the non-structural proteins of various enteroviruses [9,10,11]. On the other hand, there has been limited success in incorporating tags into structural proteins [12,13]. In the realm of virus research, inserting a tag into the capsid can offer an invaluable tool for studying various stages of the viral life cycle, including binding, uncoating, endocytosis, and assembly. Consequently, the present study aimed to engineer a novel recombinant virus that carries a commonly used tag within the capsid protein of a representative enterovirus, EV-A71. To this end, the inserted tags should be designed to avoid interfering with the essential functions of the capsid, such as receptor binding and virus assembly.

Each of the three major capsid proteins (VP1, VP2, and VP3) of EV-A71 and other enteroviruses contains a “core” consisting of an eight-stranded antiparallel beta barrel with two flanking helices [14]. Among them, the β-folds are more conserved and involved in supporting the capsid shape and structure, while the loop region is irregular and highly variable and involved in receptor binding [15]. Previous studies conducted in the 1980s reported the production of two VP1 protein fragments when type 1 poliovirus (Sabin1 strain) was treated with trypsin. Interestingly, despite trypsin treatment, the virus retained its infectivity. Further analysis revealed that the trypsin digestion site in the Sabin1 strain is situated in the BC loop between the 99th lysine and the 100th asparagine of the capsid protein VP1. A similar trypsin cleavage can also occur in the Leon and Sabin strains of type 3 poliovirus, specifically after the 100th arginine site of capsid protein VP1 [16]. Upon replacing the VP1 residues 90–105 of the type 1 poliovirus with the corresponding region of the type 3 poliovirus, the resulting hybrid poliovirus successfully induced the production of neutralizing antibodies against types 1 and 3 in both rabbits and monkeys [17]. When the VP1 residues 91–102 of the type 1 poliovirus were replaced with 18 HIV-1-specific amino acids, the resulting poliovirus chimera was able to generate HIV-1 neutralizing antibodies [18]. These findings suggest that alterations to certain sites on the loops of enterovirus capsid protein may have little effect on the virus’s infectivity. Similarly, Qin’s group reported that the introduction of two representative nucleating peptides, NE1 and N6, between the 100th and 101st amino acid sites of the BC loop of EV-A71 VP1 could induce biomineralization under physiological conditions to improve the thermal stability of EV-A71 [19]. The successful rescue of these recombinant viruses suggests that the VP1 BC loop exposed on the EV-A71 particles can similarly accommodate insertions. Despite this, it remains unclear at present where on the EV-A71 capsid foreign tags can be inserted, what specific types of tags can be integrated without compromising viral functionality, and whether these tags can be stably transmitted.

To this end, this study designed a rapid testing system based on the EV-A71 pseudovirus, screening for sites and tag characteristics that can be introduced into the EV-A71 capsid protein. Then, the optimal insertion site and tag type were selected to prepare recombinant tagged EV-A71 virus using infectious clones, and the stable passage of the inserted tags was verified. We think that the recombinant EV-A71 capsid protein carrying commonly used tags obtained in this study will provide key research tools for the future study of the various events of the enterovirus life cycle (such as cell binding, entry, uncoating, assembly, etc.). It may also be used for rapid purification and concentration of the virus and can also be useful for immunological/vaccinological studies to monitor the epitope-specific responses.

## 2. Materials and Methods

### 2.1. Materials and Reagents

Human rhabdomyosarcoma (RD) cells and human embryonic kidney cells (HEK293T) were cultured in Dulbecco’s Modified Eagle Medium (DMEM, Thermo Fisher Scientific, Waltham, MA, USA) containing 10% fetal bovine serum (FBS, Thermo Fisher Scientific, Waltham, MA, USA), 100 U/mL penicillin, and 100 µg/mL streptomycin. All cell lines were from the Chinese Academy of Sciences cell bank (Shanghai, China). pcDNA6.0-EV-A71-EGFP-Capsid and p-EV-A71-Luciferase-Replicon were provided by Professor Li Wenhui from the Beijing Institute of Life Sciences.

Mouse polyclonal antibodies against EV-A71 capsid protein VP1 and EV-A71 non-structural protein 2C were synthesized by Suzhou Unovo. The mouse anti-dsRNA antibody (SCICONS, Budapest, Hungary), rabbit anti-S antibody (SinoBiological, Shanghai, China), and mouse anti-V5 antibody (Abclonal, Wuhan, China) were purchased for this study.

The experiments conducted in this study were executed within a Biosafety Level 2 (BSL-2) laboratory, strictly adhering to the established biosafety protocols.

### 2.2. Construction of EV-A71 Capsid Plasmid with Foreign Tags

The tags were introduced using PCR primers (Tsingke, Beijing, China) in a seamless cloning method. Specifically, the pcDNA6.0-EV-A71-EGFP-Capsid was used as a DNA template for PCR amplification using the ClonExpress II One Step Cloning Kit C112 (Vazyme, Nanjing, China). The amplified PCR products were purified from agarose gel and transferred into competent DH5α cells (Tsingke, Beijing, China). After incubation at 37 °C overnight, single colonies were selected for DNA sequencing. The detailed primers for clone construction can be found in Table S1.

### 2.3. Pseudovirus Packaging

The pseudoviruses were produced by sequential transfection of capsid plasmid and Replicon RNA into HEK293 T cells as described previously [20]. The p-EV-A71-Luciferase-Replicon plasmid was linearized with the Smal I restriction enzyme (New England Biolabs, Ipswich, MA, USA), and the linearized vector was transcribed into mRNA using the T7 transcription reagent kit (Novoprotein, Suzhou, China) according to the instructions. The quality of RNA was confirmed by agarose gel electrophoresis. Capsid plasmid was first transfected into HEK293T cells at 60–80% confluence. Then, 24 h later, Replicon RNA was then transfected using lipofectamine 3000 (Thermo Fisher Scientific, Waltham, MA, USA). Pseudovirus was harvested 24 h post-RNA transfection with three rounds of freeze-thaw cycle.

The replication activity of pseudovirus in cells can be characterized by the expression levels of the inserted luciferase. Specifically, 50 μL of EV-A71 pseudovirus was mixed with 50 μL of fresh 2% FBS cell culture medium and applied to cells in a 96-well plate. After 12 h, the cell supernatant was removed and washed twice with PBS. The viral replication efficiency was analyzed by adding substrate to detect intracellular luciferase using the Luciferase Assay System (Promega, Madison, WI, USA).

### 2.4. Construction of Recombinant EV-A71 Infectious Clone

According to the manufacturer’s instructions, using the ClonExpress II One Step Cloning Kit (Vazyme, Nanjing, China), the corresponding primers were designed to clone the S (KETAAAKFERQHIDS) and V5 (GKPIPNPLLGLDST) tags into the VP1 BC loop (between residues 100 and 101) of the full-length infectious cDNA clone of clinical EV-A71 C4 strain (strain AH08/06). The primer sequences for infectious clone construction can be found in Table S2. Sequencing was used to verify whether the tag has been inserted in the recombined EV-A71 infectious clone.

### 2.5. Detection of Recombinant Virus Tags

After viral incubation, the cell culture medium was discarded, and the cells were washed three times with PBS. Viral RNA extraction and purification were performed by using the QIAamp Viral RNA Mini Kit (Qiagen, Hilden, Germany). Total RNA was obtained and used as a template for reverse transcription PCR to synthesize cDNA. Then the cDNA was used as a DNA template for PCR amplification with specific PCR primers. All PCR primers can be found in Table S3. The resulting 200 bp PCR product was sequenced to confirm that the rescued virus carries the corresponding epitope tag.

### 2.6. Plaque Assay

The titer of the virus was determined by plaque assay as previously described [21]. Twelve hours before the experiment, RD cells were seeded onto 12-well plates. The viruses were serially diluted 10-fold and added to the cells. The plates were gently shaken every 15 min and incubated at 37 °C for 2 h. After incubation, the cells were washed twice with PBS and then loaded with 2 × DMEM (4% FBS) mixed with an equal volume of 2.4% Avicel (IMCD, Shanghai, China). The plates were further incubated at 37 °C for an additional 2–3 d. To visualize the infected cells, the plates were fixed with a 1:1 mixture of 4% paraformaldehyde and 5% crystal violet staining solution for 2 h and stained with 1% crystal violet for another 1 h. Subsequently, the plates were rinsed with running water, and photographs were taken for further analysis.

### 2.7. Quantification of Viral RNA

After viral incubation, the cell culture medium was discarded, and the cells were washed three times with PBS. Viral RNA extraction and purification were performed by using the QIAamp Viral RNA Mini Kit (Qiagen, Hilden, Germany). Total RNA was obtained and used as a template for quantitative reverse transcription PCR using the HiScript II One Step RT-qPCR SYBR Green Kit (Vazyme, Nanjing, China) with virus-specific qPCR primers. The viral RNA was quantified based on a standard curve, allowing for the calculation of viral copy numbers. All qPCR primers can be found in Table S4.

### 2.8. Immunoblotting

The infected cells were collected, lysed with 1×SDS sample buffer (NCM Biotech, SouZhou, China), and heated at 100 °C for 10 min. The cell lysates were separated through a 10% SDS-PAGE gel and transferred to a PVDF membrane. The membrane was blocked with 5% milk and incubated overnight with primary antibodies at 4 °C. The membrane was then washed three times in 0.1% Tween-20/PBS and incubated with HRP-conjugated secondary antibodies (Yeason, Shanghai, China) for 2 h at room temperature. The immunoblots were visualized with the NcmECL Ultra-sensitive ECL chemiluminescent reagent kit (NCM Biotech, Suzhou, China) and observed using the Odyssey FC Imaging System (v.3.1.).

### 2.9. Flow Cytometry

RD cells were plated in a 24-well plate, with 2 × 10^5^ cells per well, and incubated overnight. The cells were then infected with tagged-rEV-A71 and parental rEV-A71 at an MOI = 1 for 6 h. After infection, the supernatant was discarded, and the cells were washed three times with PBS and then digested with trypsin. Each well was then added with 100 μL BD Cytofix/Cytoperm Fixation at 4 °C for 30 min. Following fixation, the cells were washed twice with 500 μL of 1 × BD Perm/Wash™ buffer for 5 min each. Virus dsRNA antibody or tag antibodies were then added and incubated for 30 min at room temperature in the dark, followed by two washes with 500 μL of 1 × BD Perm/Wash™ buffer for 5 min each. Finally, the cells were stained with 100 μL of diluted Donkey anti-Mouse IgG (H + L) secondary antibody or Donkey anti-Rabbit IgG (H + L) secondary antibody (Invitrogen, Carlsbad, CA, USA) in the dark for 30 min. The fluorescence signal from single cells was detected using the BD LSRFortessa™ instrument (BD LSRFortessa™ instrument (BD Biosciences, San Jose, CA, USA)). The experimental results were analyzed using flowjo software version 10.8.1.

GraphPad Prism software version 8.0.2.

### 2.10. Immunofluorescence Assay

RD cells were seeded on glass slides and incubated overnight, then continuously infected for 6 h with rEV-A71-VP1-S, rEV-A71-VP1-V5, and parental rEV-A71 virus at MOI = 1. After infection, the cells were washed twice with precooled PBS and fixed with 4% paraformaldehyde (Sigma, St. Louis, MO, USA) for 15 min. After permeabilization with 2% FBS containing 0.05% Triton X-100, the cells were stained with dsRNA or tag antibodies for 2 h in the dark. The cells were then washed with PBS twice and incubated with Alexa Fluor 594-conjugated secondary antibodies (ZSGB-BIO, Beijing, China) for 30 min, followed by washing three times. Finally, the coverslips were applied to mounting medium with DAPI (Abcam, Cambridge, UK) and analyzed using the EVOS ^®^ FL Color Imaging Systems (Life Technologies, Carlsbad, CA, USA).

### 2.11. Antibody Neutralization Experiment

RD cells were plated in a 24-well plate, with 2 × 10^5^ cells per well, and incubated overnight. When the cell density was about 70%, the recombinant viruses rEV-A71-VP1-S, rEV-A71-VP1-V5, and parental rEV-A71 were incubated with their corresponding labeled antibodies (5–10 μg/mL) at room temperature for 30 min, then added to the RD cells. After incubation at 37 °C for 6 h, the supernatant was removed, and the cells were washed twice with PBS before adding fresh culture medium. The cells were then stained with dsRNA antibody, and the viral infection rate was detected using flow cytometry.

### 2.12. Stability Analysis of Foreign Tags in Recombinant Viruses

rEV-A71-VP1-S, rEV-A71-VP1-V5, and parental rEV-A71 viruses were continuously passaged in RD cells for 15 times. Viral RNA was extracted from the 5th, 10th, and 15th generations and used as a template for RT-PCR analysis. The resulting 200 bp PCR product was subjected to 1% agarose gel electrophoresis, and after verification as a single band, it is purified and then sequenced to verify the genetic stability of the inserted tag.

### 2.13. Molecular Dynamics Simulation Protocols and Analysis

The crystal structure of the EV-A71 capsid protein (PDBID: 6I2K) and the NMR structure of 12-mer heparin (PDBID: 1HPN) were downloaded from the Protein Data Bank. Structures of EV-A71 VP1-VP4 tetramers with different tag insertions were predicted by AlphaFold2 [22] and then superimposed onto the capsid protein structure using PyMOL [23] to create VP1-tag pentamer structures. Docking of heparin onto VP1-tag pentamer was performed by ZDOCK 3.0.2 [24], with all regions except the BC, DE, and HI loops blocked during docking. The top-ranked docking result was used as the initial structure for molecular dynamics (MD) simulations.

The CHARMM-GUI webserver [25,26] and CHARMM36m force field [27] were used to generate the systems for MD simulations. The systems were solvated by TIP3 water molecules with a minimal margin of 20 Å from any protein or heparin atom to any edge of the water box. Sodium and chloride ions were added to neutralize the system using VMD software (VMD software version 1.5.4) [28], reaching a total concentration of ~150 mM. All simulations were conducted using Amber 2020 software [29]. Electrostatic interactions were calculated using the Particle Mesh Ewald (PME) method, and van der Waals interactions were computed with a cutoff of 12.0 Å. The SHAKE algorithm was applied to constrain all covalent bonds involving hydrogen atoms. The Langevin thermostat was employed for temperature control, while the Monte Carlo barostat was used to maintain the standard pressure of the system. Energy minimization was performed for 50,000 steepest descent steps and 50,000 conjugate gradient steps, where all atoms could move. Each system was gradually heated to 50 K and then to 250 K, followed by an equilibration stage at 300 K using the NVT ensemble. Production simulations were performed with the NPT ensemble for 200 ns with a timestep of 2 fs. Positional restraints were applied to backbone atoms except the BC, EF, and GH loops to maintain the relatively stable conformation of the VP1-tag pentamers, with the restraint force gradually reduced from 100 kcal·mol^−1^·A^−2^ during heating to 10 kcal·mol^−1^·A^−2^ during production. The trajectories were saved every 0.1 ns for analysis. Finally, VMD software was used to calculate the binding surface area and the contact frequency between heparin and positively charged residues on VP1.

### 2.14. Statistical Analysis

Statistical calculations were performed using GraphPad Prism software. Data are presented as mean ± SD for experiments performed with at least three replicates. The differences between two groups were analyzed using Student’s *t*-test, and multiple comparisons were performed using two-way analysis of variance (ANOVA). * represents *p* < 0.05; ** represents *p* < 0.01; and *** represents *p* < 0.001.

## 3. Results

### 3.1. Establishing a Pseudovirus System to Select the Insertion Sites and Types of Tags

**Figure 1:**
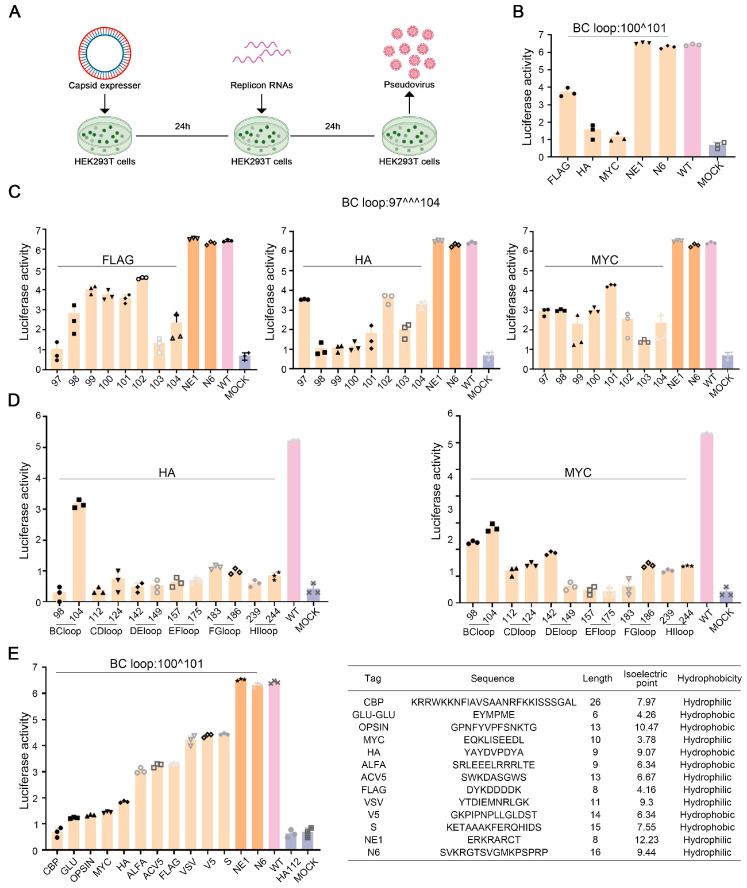
Establishment of a pseudovirus system to screen insertion sites and types of tags. (**A**) Schematic diagram of pseudovirus packaging experiment. (**B**) Activity detection of pseudoviruses introducing the common tags FLAG, HA, MYC, and exogenous peptides NE1 and N6 to the 100th site of the EV-A71 VP1 BC loop region. (**C**) Activity detection of pseudoviruses introducing tags FLAG, HA, and MYC to the 97–104th sites in the entire BC loop region of EV-A71 VP1. (**D**) Activity detection of pseudoviruses introducing tags HA and MYC to the N-terminal or C-terminal ends of various loop regions (BC, CD, DE, EF, FG, and HI) of the EV-A71 capsid protein VP1. (**E**) Activity detection of pseudoviruses introducing other small tags to the 100th site of the EV-A71 VP1 BC loop region (left table). Comparison analysis of the size, isoelectric point, and hydrophobicity of all introduced tag sequences (right panel).

We first tested three commonly used tags of HA, MYC, and FLAG introduced after the 100th site of the EV-A71 VP1 BC loop region. We generated pseudoviruses through sequential transfection of the capsid plasmid and Replicon RNA into HEK293T cells (Figure 1A and Figure S1A–C). Compared to the wild-type (WT) or positive control (NE1 and N6), the activity of the packaged pseudovirus significantly decreased (Figure 1B and Figure S1D,E). We then constructed 24 types of pseudoviruses with HA, MYC, and FLAG tags inserted at all the 97–104th sites in the entire BC loop region of EV-A71 VP1. It was found that introducing different tags at the same site or introducing the same tags at different sites had varying effects, but introducing tags all significantly reduced viral activity (Figure 1C). Next, we made pseudoviruses with HA and MYC tags introduced at the N-terminal or C-terminal ends of various loop regions (BC, CD, DE, EF, FG, and HI) of the EV-A71 capsid protein VP1. Compared to the WT, all insertions significantly diminished pseudovirus activity. However, it was observed that only pseudoviruses with tags inserted into the BC loop region exhibited relatively higher activity, whereas the activity of pseudoviruses with tags inserted into other loop regions was considerably poorer than that of the BC loop (Figure 1D).

Therefore, having confirmed the best activity associated with the insertion at the BC loop, we proceeded to test other commonly utilized tags after the 100th site of the EV-A71 VP1 BC loop. Although most tags significantly impact the pseudovirus activity, the pseudovirus infectivities associated with the three tags, namely S, VSV, and V5, are amongst the highest observed. However, it is noteworthy that their activity remains significantly lower compared to that of the WT and positive controls (Figure 1E). We analyzed the size, isoelectric point, and hydrophobicity of all introduced tag sequences. The two control peptides, NE1 and N6, that do not affect the replication activity of the pseudovirus are hydrophilic with positive charges. Among all the tags, the two of the three tags with the highest activity, S and V5, are neutral and hydrophobic. Although FLAG and MYC are hydrophilic like NE1 and N6, they are negatively charged. Judging from the pseudovirus activity, insertion of FLAG or MYC greatly affects virus infectivity (Figure 1E).

### 3.2. Constructing Infectious Clones Based on Pseudovirus Screening Results

**Figure 2:**
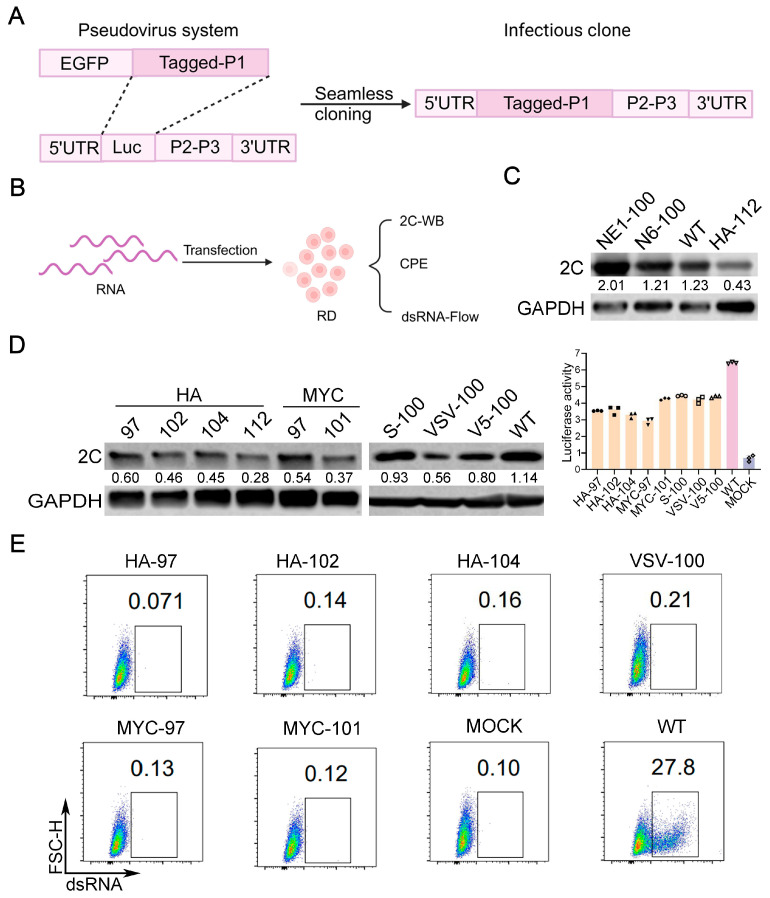
Construction of infectious clones based on the screening results of pseudoviruses. (**A**,**B**) Schematic diagram of the construction and reverse genetics rescue of recombinant EV-A71 infectious clones. (**C**,**D**) RD cells were transfected with tagged viral RNA for 24 h. After transfection, cells were lysed, and EVA71-2C” to “EV-A71 2C protein” was determined by Western blotting. The values on the graph represent the expression levels of viral 2C protein normalized by GAPDH. (**E**) RD cells were infected with recombinant viruses EV-A71-HA-97, EV-A71-HA-102, EV-A71-HA-104, EV-A71-MYC-97, EV-A71-MYC-101, and EV-A71-VSV-100 for 48 h, and viral dsRNA was detected by flow cytometry, with MOCK as a blank control.

Based on the results of the insertion screening, we attempted to rescue those recombinant enteroviruses with higher pseudovirus activity. First, we constructed the infectious clone carrying the foreign tag of the recombinant EV-A71 through seamless cloning (Figure 2A). The infectious clone was then transcribed into viral RNA in vitro and then transfected into RD cells to collect the culture supernatant to verify the viability of the obtained recombinant virus (Figure 2B). We selected the pseudovirus with the highest infectivity (insertion of NE1 and N6 at the VP1 100 site) as the positive control and no activity (insertion of HA at the VP1 112 site) as the negative control. After the viral RNAs were transfected into RD cells, we first examined the expression level of virus proteins by Western blotting. For NE1 and N6 pseudoviruses with similar activity to the WT pseudovirus, the rescued recombinant virus has a similar expression level of virus protein 2C with the WT. In contrast, for insertion of HA at the VP1 112 site, the recombinant showed a significantly lower expression level of virus protein 2C (Figure 2C). Thus, the results indicated that the activity of the pseudovirus tests reflects the potential vitality of rescuing the recombinant virus. According to the results of the pseudovirus test, we also selected BC loop 97, 102, and 104 sites to introduce HA; BC loop 97 and 101 sites to introduce MYC; “the BC loop 100 site to introduce S, VSV and V5 to construct infectious clones”. We first examined the virus 2C protein expression levels by Western blotting (Figure 2D). We found that the 2C expression levels of infectious clones carrying foreign tags are proportional to the activity of pseudoviruses. Specifically, the introduction of S and V5 epitopes exhibited the highest levels of expression (Figure 2D).

To rescue the viable recombinant viruses, we collected the culture supernatants two days following viral RNA transfection in RD cells, as the P0 generation virus (named as rEV-A71-tag-site). Subsequently, these P0 viruses were serially passaged in RD cells to examine the appearance of cytopathic effect (CPE) and to amplify the recombinant virus. It was observed that only the recombinant virus incorporating S and V5 tags at the VP1 100 site exhibited CPE, whereas the others were inviable. We designated the recombinant virus displaying CPE as rEV-A71-VP1-S and rEV-A71-VP1-V5.

Viruses without CPE may not firmly indicate the failure of rescue, for example, the recombinants may have weaker virulence and do not cause CPE. To verify, we infected cells with culture supernatant from those clones, which failed to induce CPE (EV-A71-HA-97, EV-A71-HA-102, EV-A71-HA-104, EV-A71-MYC-97, EV-A71-MYC-101, and EV-A71-VSV-100) and examined potential virus infectivity (indicated by dsRNA) by flow cytometry. The results confirmed that those recombinant EV-A71 clones are not viable (Figure 2E).

### 3.3. Virological Characteristics of rEV-A71-S and rEV-A71-V5

**Figure 3:**
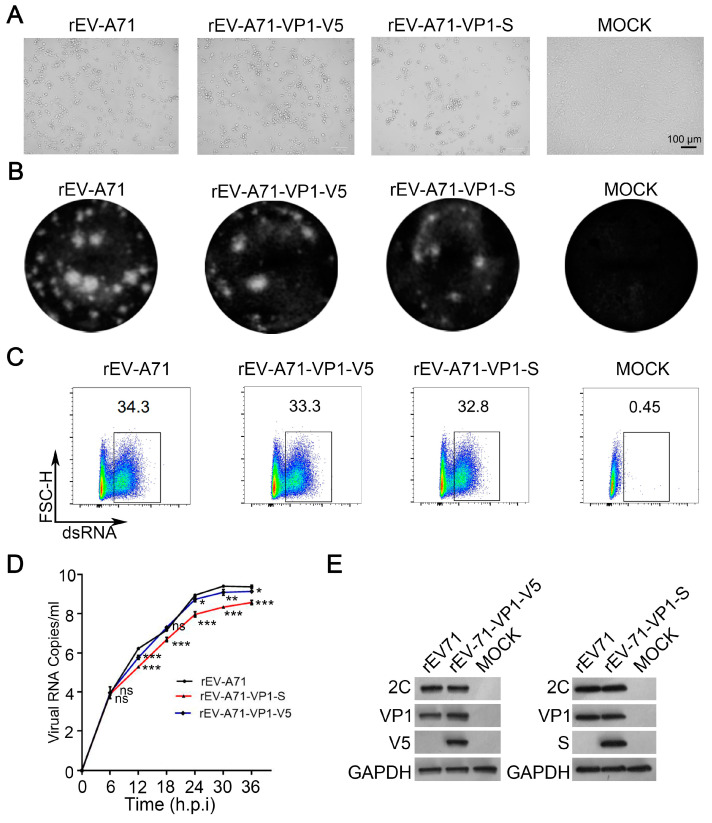
Virological characteristics of recombinant viruses rEV-A71-S and rEV-A71-V5. (**A**) RD cells were transfected with rEV-A71-VP1-S, rEV-A71-VP1-V5, and parental rEV-A71 viral mRNA, and cytopathic effect was observed after 24 h, with MOCK as a blank control. (**B**) Plaque morphology of rEV-A71-VP1-S, rEV-A71-VP1-V5, and parental rEV-A71 virus after infection of RD cells, with MOCK as a blank control. (**C**) RD cells were infected with rEV-A71-VP1-S, rEV-A71-VP1-V5, and parental rEV-A71 at an MOI of 1 for 6 h, and viral dsRNA was detected by flow cytometry, with MOCK as a blank control. (**D**) One-step growth curves of rEV-A71-VP1-S, rEV-A71-VP1-V5, and parental rEV-A71. RD cells were infected with rEV-A71-VP1-V5, rEV-A71-VP1-S, and parental rEV-A71 at an MOI of 0.1. At 6, 12, 18, 24, 30, and 36 h, the viral genomic RNA was extracted and then measured by qPCR. Statistical differences were determined by *t*-test. * represents *p* < 0.05; ** represents *p* < 0.01; and *** represents *p* < 0.001. Data are means ± SD of three replicate samples. (**E**) RD cells were infected with rEV-A71-VP1-S and parental rEV-A71 at an MOI of 0.1 for 12 h. After infection, cells were lysed, and EVA71-2C, VP1, V5, and S were determined by Western blotting, with MOCK as a blank control.

rEV-A71-VP1-S, rEV-A71-VP1-V5, and WT EV-A71 caused clear CPE in RD cells 24 h after infection (Figure 3A). Plaque assays indicated that the plaque sizes of rEV-A71-VP1-S, rEV-A71-VP1-V5, and WT EV-A71 viruses were comparable (Figure 3B). However, a reduced number of plaques suggests that the inserted tag may still have a certain influence on the virus’s activity. We then conducted a growth kinetics test to measure the viral infectivity. Using MOI = 1 of rEV-A71-VP1-S, rEV-A71-VP1-V5, and WT EV-A71, we found through flow cytometry that the infection rate of the recombinant virus was similar to that of the WT virus (Figure 3C). To identify potential differences between rEV-A71-VP1-S, rEV-A71-VP1-V5, and WT EV-A71, we further examined the one-step growth curve of these viruses in RD cells. Despite the results indicating that, as time progressed, the growth rates of both recombinant viruses were slightly yet significantly lower in comparison to the WT EV-A71 virus, all virus strains exhibited a clearly defined exponential growth pattern in a similar fashion. This observation suggests that the incorporation of the S and V5-tag into VP1 has minimal impact on the viral activity within RD cells (Figure 3D). To further validate whether the inserted epitope could be accurately detected, we utilized Western blotting to analyze the viral proteins. Our findings confirmed that rEV-A71-VP1-S could be successfully detected using an S tag antibody, while rEV-A71-VP1-V5 could be detected with a V5 tag antibody (Figure 3E).

### 3.4. Applications and Functional Verification of Recombinant Virus Labels

**Figure 4:**
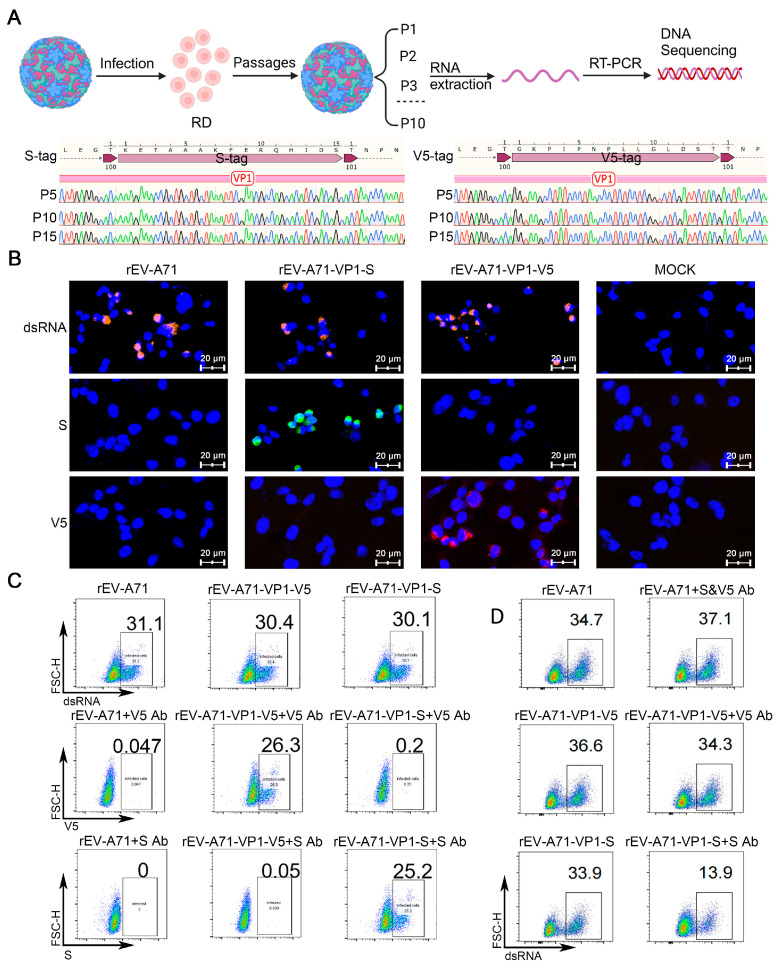
Application and functional validation of recombinant virus tags. (**A**) Verification of genetic stability of tags. Sequencing of the viral genome of the fifth, tenth, and fifteenth generations of rEV-A71-VP1-S and rEV-A71-VP1-V5 and comparison analysis using SnapGene of the foreign tag sequencing results. (**B**) RD cells were infected with rEV-A71-VP1-S, rEV-A71-VP1-V5, and parental rEV-A71 at an MOI of 1 for 6 h, and viral dsRNA, V5, and S were detected by fluorescence microscopy, orange for dsRNA labeling, green for S labeling, red for V5 labeling, and blue for cell nucleus (DAPI), with MOCK as a blank control. (**C**) RD cells were infected with rEV-A71-VP1-S, rEV-A71-VP1-V5, and parental rEV-A71 at an MOI of 1 for 6 h, and viral dsRNA, V5, and S were detected by flow cytometry. (**D**) Neutralizing antibody experiment. Recombinant virus rEV-A71-VP1-V5 with V5 tag antibody, rEV-A71-VP1-S with S tag antibody, and parental virus rEV-A71 with V5 and S tag antibodies were incubated at room temperature for 30 min, then infected RD cells for 6 h, followed by staining with dsRNA antibody. Virus infection rate was detected by flow cytometry.

To determine the genetic stability of the recombinant tagged virus in vitro, we cultured the recombinant viruses rEV-A71-VP1-S and rEV-A71-VP1-V5 in RD cells for 15 consecutive passages, and after the 5th, 10th, and 15th passages, we extracted the virus RNA for RT-PCR analysis and sequencing. Even after 15 consecutive passages in vitro, the inserted tags remained stable during the passage in RD cells (Figure 4A).

The aforementioned protein immunoblotting confirmed the specificity of the epitope. Next, we wondered if an epitope-specific antibody could enable the detection of infected viruses through immunofluorescence and flow cytometry assays. Indeed, the rEV-A71-VP1-V5 recombinant was specifically recognized by the V5 tag antibody, and the rEV-A71-VP1-S was specifically recognized by the S tag antibody (Figure 4B,C). The immunofluorescent image revealed that all the virus capsid proteins were distributed in the cytoplasm, aligning with the known intracellular location of the virus.

Given that the inserted epitope within the VP1 BC loop is exposed externally on the viral particles, we were curious whether the binding of epitope antibody to the tagged viruses might neutralize the viral infectivity. Thus, we conducted an antibody neutralization experiment, and the results clearly showed that the S-specific antibody can partially neutralize the recombinant virus rEV-A71-VP1-S. As controls, the presence of S or V5-specific antibodies did not display any neutralizing effects on the WT rEV-A71 (Figure 4D). These results indicate that the inserted epitope is likely located away from the virus–host cell interface. Furthermore, the use of epitope-specific antibody does not interfere with viral activity and is compatible with assays for various viral life cycle events.

### 3.5. Molecular Dynamics Simulations for Tagged EV-A71 and Heparin

**Figure 5:**
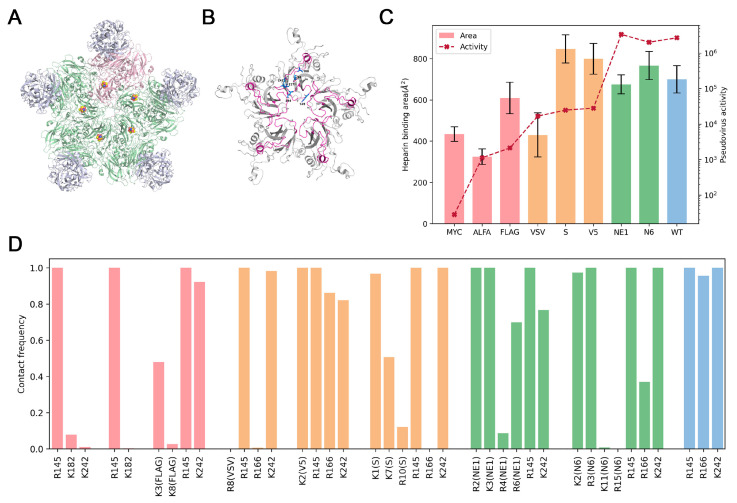
Structural insights into the impacts of tag insertion on EV-A71–heparin interactions. (**A**) Structure of EV-A71-SCARB2 complex (PDBID: 6I2K). SCARB2s are colored in gray, and EV-A71 capsid protein is colored in pale green and pale pink to distinguish the interfaces between VP1 and VP4 tetramers. Sites 100 and 101 are shown as spheres. (**B**) The VP1-tag pentamer used for heparin docking and MD simulations. The previously reported binding sites are shown as marine sticks. The BC, DE, and HI loop regions, which were not blocked in docking and allowed free movement during MD simulations, are colored in magenta. (**C**) Comparison of EV-A71–heparin binding surface area during the 100–200 ns simulations, where the overall conformations were relatively stable. (**D**) Comparison of contact frequency between heparin and positively charged residues on VP1 during the 100–200 ns simulations.

Finally, we aim to comprehend the factors that influence the creation of viral recombinants with epitope tags on the capsid proteins. To this end, the inserted epitope must not disrupt the functions of the capsid, such as receptor binding and virus assembly. In fact, the structure analysis of the EV-A71 capsid revealed that, among the various loop regions of VP1, the BC loop is centrally positioned and does not directly participate in the interactions between viral capsid proteins. Furthermore, the 100th amino acid site on VP1, situated at the apex of the BC loop and facing the canyon region, is distant from any potential interfaces critical for virus assembly or virus interactions with its receptor SCARB2 (Figure 5A and Figure S4). Next, we aim to delve into the reasons why not all epitopes inserted at this particular site resulted in the generation of viable recombinant viruses. Since the BC loop is near the five-fold axis, which is the primary binding region of the EV-A71 attachment receptor, heparan sulfate [30,31]. We speculated that the insertion of tags might affect heparin binding, thereby impacting viral activities. To investigate this, we modeled various VP1-tag pentamer structures, performed molecular docking to heparin, and ran 200 ns MD simulations of the complexes. Since all the previously reported heparin binding sites are on the BC, DE, and HI loop regions, residues from the other regions were blocked during docking and constrained during MD simulations (Figure 5B). We calculated the binding surface area between VP1 pentamers and heparin, which was 699.7 Å^2^ for the wild-type. For the negative-tagged types, the binding area decreased significantly: 324.4 Å^2^ for ALFA, 434.3 Å^2^ for MYC, and 609.2 Å^2^ for FLAG, suggesting that tag insertion substantially weakened the interaction with heparin. In comparison, the binding area of the positive-tagged types was equivalent to (NE1 and N6) or larger than (V5 and S) that of the wide type (Figure 5C).

Electrostatic interactions are key molecular determinants of the EV-A71-heparan sulfate interaction [32]. To elaborate on the impacts of different tag insertions on the interaction pattern, we analyzed the contact frequency between positively charged residues on VP1 and heparin throughout the simulation trajectories (Figure 5D and Figure S2). We found that in the wild-type EV-A71 VP1, residues 145, 166, and 242 were critical and stable heparin-binding sites. The steric hindrance introduced by tag insertion after the 100th site disrupted heparin interactions with these three sites, but positive tags provided additional positively charged residues as compensations (Figure S3). We further identified that these important compensatory residues were typically at the beginning of the tags, exhibiting more stable heparin binding compared to those in the middle or end positions. For example, the contact frequency of R2 and K3 on NE1 with heparin was 100%, much higher than that of R4 (8.6%) and R6 (69.9%). K1 at the beginning of the S tag also revealed a higher contact frequency than K7 (50.7%) and R10 (12.1%). This disparity can likely be ascribed to the more stringent conformational constraints imposed by the surrounding regions on the residues at the beginning of the inserted tags.

## 4. Discussion

In this study, we engineered two infectious clones of recombinant EV-A71, each incorporating a commonly utilized epitope tag on its capsid protein, and successfully generated viable recombinant viruses. It was discovered that the recombinant virus exhibits similar virological characteristics to the parent strain, and the tag persists stably even after multiple passages in the infected cell line.

To streamline the identification of potential sites and compatible tag types for generating functional recombinant viruses, we implemented a novel screening approach. This involved engineering capsid plasmids with various tags on the EV-A71 VP1 protein and co-transfecting them with viral Replicon RNA into HEK293T cells to produce pseudoviruses. Utilizing a single-round infection system, we efficiently and quantitatively assessed the effect of the inserted tags on viral activity. This process is significantly more straightforward and faster than creating EV-A71 infectious clones with foreign tags. The experimental outcomes provided deeper insights, enabling us to classify different tagged viruses based on their luciferase activities. With these quantitative data, we could then select insertion sites that exhibited higher activity and proceed to rescue the virus. This strategy enhances the success rate and conserves time and resources.

We conducted extensive testing on the insertion of tags at all sites of the VP1 BC loop of the EV-A71 capsid protein, as well as at the C- and N-termini of various other loop regions, with the aim of identifying optimal sites capable of accommodating foreign tags. Unfortunately, no additional viable sites were identified within the VP1 loop region. Furthermore, our attempts to insert a range of commonly used tags after the 100th site of the VP1 BC loop yielded limited success, with only the S and V5 tags being successfully incorporated. We observed that some recombinant viruses, such as rEV-A71-VP1-VSV, are non-viable, whereas their corresponding pseudoviruses showed certain activity as seen in rEV-A71-VP1-S or rEV-A71-VP1-V5, indicating notable distinctions between pseudovirus and authentic virus assays. In the context of a pseudovirus system, the capsid protein and the replicating genome are packaged into particles via trans-complementation. If alterations to the capsid protein do not impede assembly and infection, the pseudovirus can successfully form and function. However, authentic viral infections require that modifications to the capsid protein also do not disrupt genome replication, rendering this process more stringent.

In order to gain a deeper understanding of the principles governing the placement of tags in the engineering of recombinant enteroviruses, we modeled the structures of the EV-A71 capsid and conducted a thorough analysis of the potential impact of foreign epitopes on receptor binding. The structure of EV-A71 in complex with its receptor SCARB2 protein revealed that the 100th site on the VP1 BC loop is distant from the EV-A71-SCARB2 binding interface and not implicated in capsid assembly. As a result, the insertion of a tag of certain length at this site is anticipated to have a negligible effect on viral activity. Nevertheless, the attachment of a relatively lengthy CBP tag may interfere with other regions, thereby disrupting the assembly of the capsid proteins and leading to the lowest pseudovirus activity among all tested tags. Future endeavors to engineer tagged enteroviruses at alternative sites or in different strains could benefit from the insights gained from this analysis.

Regarding the analysis of the type of tags inserted after the 100th site, it has been reported that inserting peptides NE1 and N6 at the same sites can augment virus stability. We conducted a comparative analysis of the physicochemical properties of the exogenous peptides and tags, revealing that the peptides NE1 and N6 are negatively charged and hydrophilic, while the S and V5 tags are neutral and hydrophobic. Given that the VP1 residues, located around the five-fold axis of both EV-A71 and CVA16 viruses, play a pivotal role in mediating interactions with heparan sulfate, we hypothesized that the insertion of tags at the VP1 BC loop might disrupt the viral interaction with its attachment receptor, heparan sulfate. We conducted additional MD simulations, and the data we obtained further supported this hypothesis. Based on the experimental results and structural analysis, we propose the following assumptions for tags that preserve recombinant virus activities: (1) the tag should be of appropriate length to avoid direct interference with other regions and capsid protein assembly, and (2) the initial part of the tag should contain positively charged residues, with minimal adjacent acidic residues, as they might shield electrostatic interactions and repel heparin, which carries a high density of negative charge. We believe that the rules outlined herein have the potential to be extended for the identification of compatible sites capable of accommodating foreign epitopes, thereby meriting further testing in the future.

The recombinant enterovirus, engineered by genetically altering the EV-A71 capsid protein to incorporate foreign tags, will offer novel tools for various enterovirus research endeavors. For instance, the introduced tag can bind with exceptional specificity to its corresponding antibody. This enables the utilization of tag antibody-conjugated beads (such as S and V5) to facilitate the purification of the tagged virus or to employ the antibody in the process of capturing viral particle-interacting molecules. The tagged EV-A71 capsid may also serve as a beneficial tool for immunology and vaccinology studies. For instance, following vaccination with the genetically engineered, tagged viruses, immune responses specific to the epitopes can be monitored with convenience. Furthermore, the tagged recombinant EV-A71 capsid offers valuable tools for investigating pivotal stages of the EV-A71 lifecycle, such as virus-cell binding, cellular entry, intracellular trafficking, uncoating, and capsid assembly. Additionally, the epitope tags on EV-A71 can be employed in immunohistochemistry studies within animal infection models.

## 5. Conclusions

In summary, we implemented a novel screening strategy that involved assessing the activity of pseudoviruses using a single-round infection system. This approach improved the process of identifying viable engineering recombinant viruses with foreign tags exposed on the viral particles. Through structural modeling and computational analysis, we have gained a deeper understanding of the compatibility and potential impacts of foreign tags on viral activity. Ultimately, we successfully created two new recombinant strains of EV-A71 stably carrying S and V5 tags. These recombinant viruses could serve as valuable tools for investigations into viral replication, host–pathogen dynamics, and immunological responses.

## Data Availability

All data generated or analyzed during this study are included in this published article.

## Supplementary Materials

The following supporting information can be downloaded at: https://www.mdpi.com/article/10.3390/v17010128/s1, Figure S1: Construction and verification of single-round infection system; Figure S2: Detailed analysis of electrostatic interactions between heparin and positively charged residues on tagged VP1 during the 100–200 ns MD simulations; Figure S3: Tagged VP1 pentamer-heparin structures after 200 ns MD simulations; Figure S4: Structure models of EV-A71-S and EV-A71-V5. Table S1: PCR primer sequences for construction of EV-A71 capsid plasmid with foreign tags; Table S2: PCR primer sequences for construction of recombinant EV-A71 infectious clone; Table S3: PCR primer sequences for detection of recombinant virus tags; Table S4: qPCR primer sequences.

## References

[1] Simmonds P., Gorbalenya A.E., Harvala H., Hovi T., Knowles N.J., Lindberg A.M., Oberste M.S., Palmenberg A.C., Reuter G., Skern T. (2020). Recommendations for the nomenclature of enteroviruses and rhinoviruses. Arch. Virol..

[2] Lei X., Xiao X., Wang J. (2016). Innate Immunity Evasion by Enteroviruses: Insights into Virus-Host Interaction. Viruses.

[3] Gundamraj V., Hasbun R. (2023). Viral meningitis and encephalitis: An update. Curr. Opin. Infect. Dis..

[4] Tschöpe C., Ammirati E., Bozkurt B., Caforio A.L.P., Cooper L.T., Felix S.B., Hare J.M., Heidecker B., Heymans S., Hübner N. (2021). Myocarditis and inflammatory cardiomyopathy: Current evidence and future directions. Nat. Rev. Cardiol..

[5] Carré A., Vecchio F., Flodström-Tullberg M., You S., Mallone R. (2023). Coxsackievirus and Type 1 Diabetes: Diabetogenic Mechanisms and Implications for Prevention. Endocr. Rev..

[6] Ooi M.H., Wong S.C., Lewthwaite P., Cardosa M.J., Solomon T. (2010). Clinical features, diagnosis, and management of enterovirus 71. Lancet Neurol..

[7] Lei D., Griffiths E., Martin J. (2020). WHO working group meeting to develop WHO Recommendations to assure the quality, safety and efficacy of enterovirus 71 vaccines. Vaccine.

[8] Li M.L., Shih S.R., Tolbert B.S., Brewer G. (2021). Enterovirus A71 Vaccines. Vaccines.

[9] Liu D., Liu C., Liu X., Li X., Huang L., Hu J., Wei Y., Zhu H., Zhang Q., Wang X. (2019). Rescue and characterization of a recombinant HY12 bovine enterovirus carrying a foreign HA epitope in the 3A nonstructural protein. Arch. Virol..

[10] van der Schaar H.M., Melia C.E., van Bruggen J.A., Strating J.R., van Geenen M.E., Koster A.J., Bárcena M., van Kuppeveld F.J. (2016). Illuminating the Sites of Enterovirus Replication in Living Cells by Using a Split-GFP-Tagged Viral Protein. mSphere.

[11] Teterina N.L., Pinto Y., Weaver J.D., Jensen K.S., Ehrenfeld E. (2011). Analysis of poliovirus protein 3A interactions with viral and cellular proteins in infected cells. J. Virol..

[12] Liu D., Liu C., Hu J., Hang L., Li X., Wei Y., Zhu H., Zhang Q., Wang X. (2018). Construction and evaluation of HA-epitope-tag introduction onto the VP1 structural protein of a novel HY12 enterovirus. Virology.

[13] Seago J., Jackson T., Doel C., Fry E., Stuart D., Harmsen M.M., Charleston B., Juleff N. (2012). Characterization of epitope-tagged foot-and-mouth disease virus. J. Gen. Virol..

[14] Plevka P., Perera R., Cardosa J., Kuhn R.J., Rossmann M.G. (2012). Crystal structure of human enterovirus 71. Science.

[15] Kobayashi K., Koike S. (2020). Cellular receptors for enterovirus A71. J. Biomed. Sci..

[16] Fricks C.E., Icenogle J.P., Hogle J.M. (1985). Trypsin sensitivity of the Sabin strain of type 1 poliovirus: Cleavage sites in virions and related particles. J. Virol..

[17] Murray M.G., Kuhn R.J., Arita M., Kawamura N., Nomoto A., Wimmer E. (1988). Poliovirus type 1/type 3 antigenic hybrid virus constructed in vitro elicits type 1 and type 3 neutralizing antibodies in rabbits and monkeys. Proc. Natl. Acad. Sci. USA.

[18] Evans D.J., McKeating J., Meredith J.M., Burke K.L., Katrak K., John A., Ferguson M., Minor P.D., Weiss R.A., Almond J.W. (1989). An engineered poliovirus chimaera elicits broadly reactive HIV-1 neutralizing antibodies. Nature.

[19] Lyu K., Wang G.C., He Y.L., Han J.F., Ye Q., Qin C.F., Chen R. (2015). Crystal structures of enterovirus 71 (EV71) recombinant virus particles provide insights into vaccine design. J. Biol. Chem..

[20] Chen P., Song Z., Qi Y., Feng X., Xu N., Sun Y., Wu X., Yao X., Mao Q., Li X. (2012). Molecular determinants of enterovirus 71 viral entry: Cleft around GLN-172 on VP1 protein interacts with variable region on scavenge receptor B 2. J. Biol. Chem..

[21] Wang M., Yan J., Zhu L., Wang M., Liu L., Yu R., Chen M., Xun J., Zhang Y., Yi Z. (2020). The Establishment of Infectious Clone and Single Round Infectious Particles for Coxsackievirus A10. Virol. Sin..

[22] Jumper J., Evans R., Pritzel A., Green T., Figurnov M., Ronneberger O., Tunyasuvunakool K., Bates R., Žídek A., Potapenko A. (2021). Highly accurate protein structure prediction with AlphaFold. Nature.

[23] Schrödinger L.D.W. (2020). PyMOL. http://www.pymol.org/pymol.

[24] Pierce B.G., Hourai Y., Weng Z. (2011). Accelerating protein docking in ZDOCK using an advanced 3D convolution library. PLoS ONE.

[25] Jo S., Kim T., Iyer V.G., Im W. (2008). CHARMM-GUI: A web-based graphical user interface for CHARMM. J. Comput. Chem..

[26] Brooks B.R., Brooks C.L., Mackerell A.D., Nilsson L., Petrella R.J., Roux B., Won Y., Archontis G., Bartels C., Boresch S. (2009). CHARMM: The biomolecular simulation program. J. Comput. Chem..

[27] Lee J., Cheng X., Swails J.M., Yeom M.S., Eastman P.K., Lemkul J.A., Wei S., Buckner J., Jeong J.C., Qi Y. (2016). CHARMM-GUI Input Generator for NAMD, GROMACS, AMBER, OpenMM, and CHARMM/OpenMM Simulations Using the CHARMM36 Additive Force Field. J. Chem. Theory Comput..

[28] Humphrey W., Dalke A., Schulten K. (1996). VMD: Visual molecular dynamics. J. Mol. Graph..

[29] Case D.A., Aktulga H.M., Belfon K., Cerutti D.S., Cisneros G.A., Cruzeiro V.W.D., Forouzesh N., Giese T.J., Götz A.W., Gohlke H. (2023). AmberTools. J. Chem. Inf. Model..

[30] Tan C.W., Sam I.C., Lee V.S., Wong H.V., Chan Y.F. (2017). VP1 residues around the five-fold axis of enterovirus A71 mediate heparan sulfate interaction. Virology.

[31] Tan C.W., Poh C.L., Sam I.C., Chan Y.F. (2013). Enterovirus 71 uses cell surface heparan sulfate glycosaminoglycan as an attachment receptor. J. Virol..

[32] Hsieh C.F., Jheng J.R., Lin G.H., Chen Y.L., Ho J.Y., Liu C.J., Hsu K.Y., Chen Y.S., Chan Y.F., Yu H.M. (2020). Rosmarinic acid exhibits broad anti-enterovirus A71 activity by inhibiting the interaction between the five-fold axis of capsid VP1 and cognate sulfated receptors. Emerg. Microbes Infect..

