# Supplementary material for: Screening of Insertion Sites and Tags on EV-A71 VP1 Protein for Recombinant Virus Construction
Source: Viruses. 2025 Jan 17;17(1):128. doi: 10.3390/v17010128 (PMC11768620; doi:10.3390/v17010128)
Supplement: Supplementary file 1 [file viruses-17-00128-s001.zip › viruses-3359011-supplementary.pdf]

viruses

MDPI

Supplementary Materials

Screening of Insertion Sites and Tags on EV-A71 VP1 Protein for Recombinant Virus Construction

Miomiao Kang [1,†], Xiangyi Li [2,†], Xiaohong Li [1], Rui Yu [3], Shuo Zhang [1], Jingjing Yan [4], Xiaoyan Zhang [4], Jianqing Xu [1], Buyong Ma [2,*] and Shuye Zhang [1,*]

[1] Clinical Center for Biotherapy, Zhongshan Hospital, Fudan University, Shanghai 200433, China; kangmiaomiao2024@163.com (M.K.); 23111210007@m.fudan.edu.cn (X.L.); 21211300001@m.fudan.edu.cn (S.Z.); xujianqing@fudan.edu.cn (J.X.)
[2] Engineering Research Center of Cell & Therapeutic Antibody (MOE), School of Pharmacy, Shanghai Jiao Tong University, Shanghai 200240, China; waterrr-lxy@sjtu.edu.cn
[3] Patronus Biotech Co., Ltd., Guangzhou 510715, China; bioyurui@163.com
[4] Shanghai Public Health Clinical Center, Fudan University, Shanghai 201508, China; yanjing872006@126.com (J.Y.); zhangxiaoyan@fudan.edu.cn (X.Z.)
* Correspondence: mabuyong@sjtu.edu.cn (B.M.); shuye_zhang@fudan.edu.cn (S.Z.)
† These authors contributed equally to this work.

Single-round infection system refers to the lack of structural genes in the genome of the virus, which can only replicate in the cell when it infects cells, cannot express capsid proteins, and cannot assemble into normal virus particles, which is called "pseudovirus". Pseudoviral packaging includes transfection of capsid plasmid and Replicon RNA as well as collection of pseudoviruses (Figure S1A). The transcriptional EV-A71 Replicator RNA in vitro was detected by agarose gel electrophoresis. It was found that the RNA bands had high specificity, main bands and no obvious degradation and dispersion. EV-A71 Replicon RNA was transfected into RD cells for 24 h to detect luciferase activity. The results showed that the replication activity value of EV-A71 Replicon RNA was within the normal range, so it could be used for the next cell transfection (Figure S1B). GFP on the capsid plasmid was used to measure transfection efficiency, and the results of transfection for 24 h were shown in Figure S1C.

In order to successfully construct a single round infection system of EV-A71 pseudovirus with exogenous label carried by capsid protein, we refer to the work of Qin et al in 2015[1]. We constructed capsid plasmids with two representative nucleating peptides NE1 and N6 inserted between the BC loop100 and 101 amino acid sites of EV-A71 capsid protein VP1 as a positive control (Figure S1D). The NE1-capsid and N6-capsid plasmids were transfected into HEK293T cells for 24 h and then the Replicon RNA was transfected. After 24 h, the supernatant was harvested and the cell fragments were removed to obtain pseudovirus with capsid protein carrying foreign peptides. The RD cells were infected with pseudoviruses with exogenous peptides to detect the luciferase activity. There was little difference in the activity of pseudoviruses with exogenous peptides compared with wild type (Figure S1E). It can be seen that the screening system is working properly and effective.

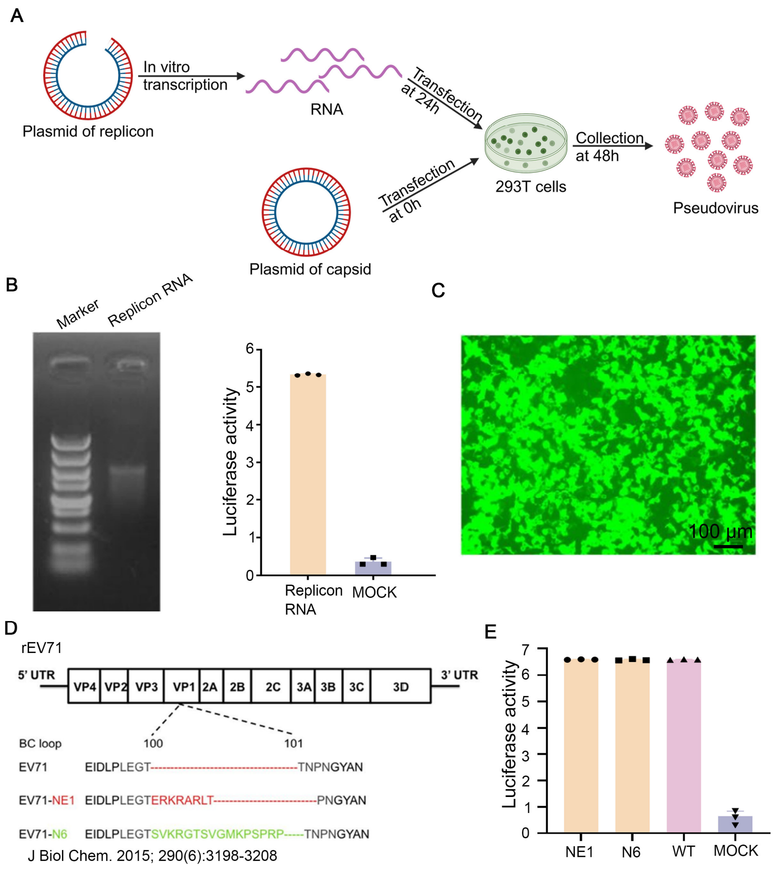

Figure S1. Construction and verification of single-round infection system. (**A**)Schematic diagram of pseudovirus packaging process. (**B**) In vitro transcription of viral Replicon RNA by agarose gel electrophoresis (left) and detection of RNA replication activity (right) M: Marker; R: Replicon RNA. (**C**) Viral capsid protein plasmid transfection results for 24 h. (**D**) Schematic diagram of pseudovirus construction with the introduction of NE1&N6 between the BC loop 100 and 101 amino acid sites of EV-A71 capsid protein VP1. (E) Detection of NE1&N6 pseudovirus and parental EV-A71 pseudovirus luciferase activity.

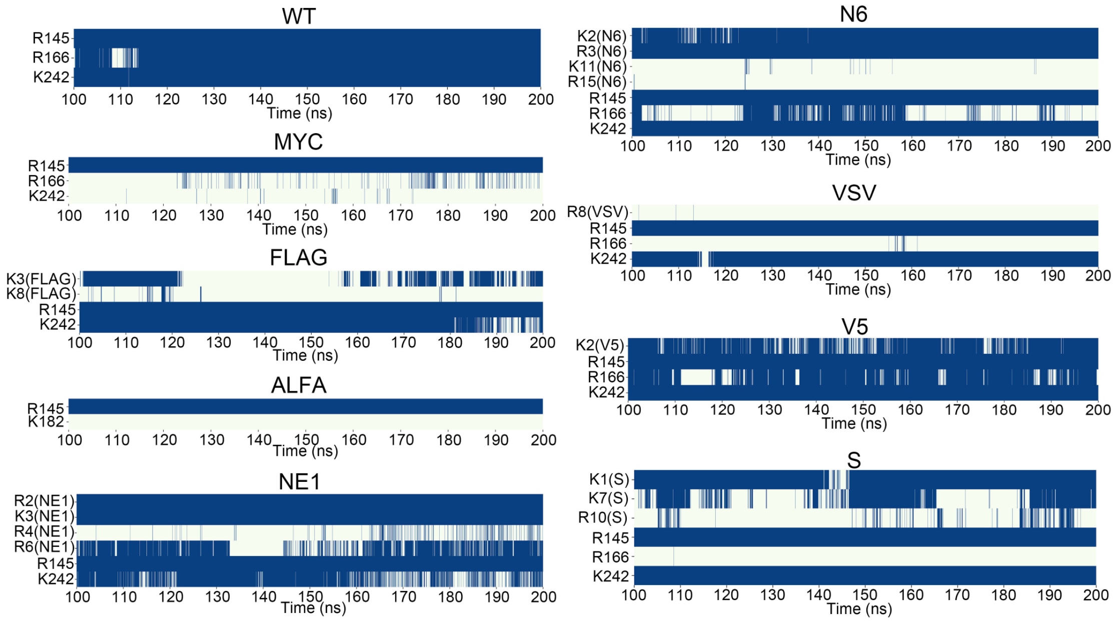

Figure S2. Detailed analysis of electrostatic interactions between heparin and positively-charged residues on tagged VP1 during the 100-200 ns MD simulations. A residue is considered to interact

with heparin if any of its atoms is within 5 Å of a heparin atom. The deepblue regions indicate sustained contact in the time period, while white regions represent there is no contact.

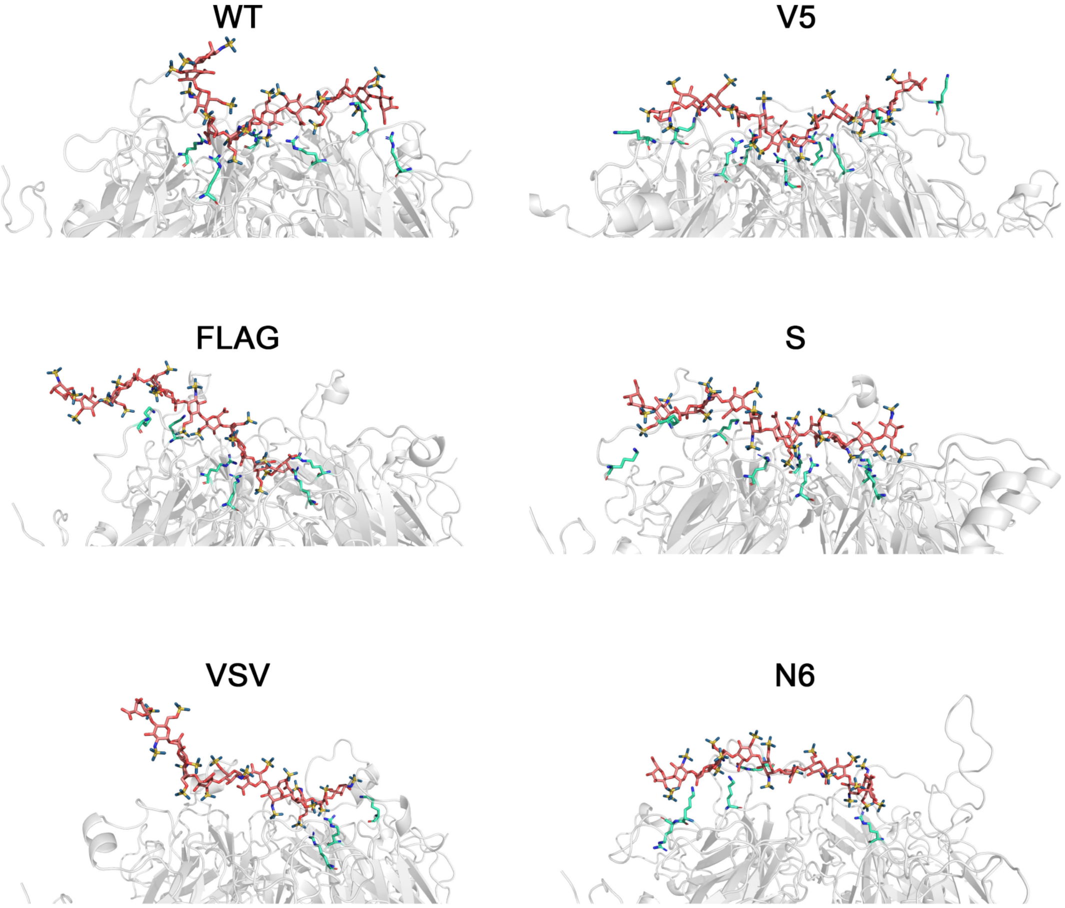

Figure S3. Tagged VP1 pentamer-heparin sturctures after 200 ns MD simulations. Heparin is shown as salmon sticks, and the positively-charged residues on tagged VP1 that interact with heparin are shown as cyangreen sticks.

Table S1. PCR primer sequences for construction of EV-A71 Capsid Plasmid with Foreign Tags.

Name		sequences (5'→3')
MYC-98	Forward	CTTCAGAGATGAGTTTCTGCTCATTTGGGTTAGTTGTGCCCTC
	Reverse	GAGCAGAAACTCATCTCTGAAGAGGATCTGGGCACAACTAACCCAAATGGT
MYC-104	Forward	CAGATCCTCTTCAGAGATGAGTTTCTGCTCCTCAAGAGGGAGATCTATCTCTCC
	Reverse	GAGCAGAAACTCATCTCTGAAGAGGATCTGGGTTATGCCAACTGGGACATAGAC
MYC-112	Forward	CAGATCCTCTTCAGAGATGAGTTTCTGCTCATTTGGGTTAGTTGTGCCCT
	Reverse	GAGCAGAAACTCATCTCTGAAGAGGATCTGATAACAGGTTACGCGCAAATGC
MYC-124	Forward	CAGATCCTCTTCAGAGATGAGTTTCTGCTCGTCTATGTCCCAGTTGGCATAACC
	Reverse	GAGCAGAAACTCATCTCTGAAGAGGATCTGCTATTCACCTACATGCGCTTTG
MYC-142	Forward	CAGATCCTCTTCAGAGATGAGTTTCTGCTCCTCTACCTTTCTACGCATTTGCGC
	Reverse	AATGAGCAGAAACTCATCTCTGAAGAGGATCTGACCGGGCAAGTTGTCCC
MYC-149	Forward	CAGATCCTCTTCAGAGATGAGTTTCTGCTCGGGTGTGCACGCAACAAAAGT
	Reverse	GAGCAGAAACTCATCTCTGAAGAGGATCTGCAATTGCTCCAATATATGTTTGTGCC AC
HA-98	Forward	CAGATCCTCTTCAGAGATGAGTTTCTGCTCTGGGACAACTTGCCCGGTGG
	Reverse	TACCCATACGATGTTCCAGATTACGCTGGCACAACTAACCCAAATGGT
HA-104	Forward	AGCGTAATCTGGAACATCGTATGGGTACTCAAGAGGGAGATCTATCTCTCCAAC

	Reverse	TACCCATACGATGTTCCAGATTACGCTGGTTATGCCAACTGGGACATAGACAT
HA -112	Forward	AGCGTAATCTGGAACATCGTATGGGTAATTTGGGTTAGTTGTGCCCTC
	Reverse	TACCCATACGATGTTCCAGATTACGCTATAACAGGTTACGCGCAAATGC
HA-124	Forward	AGCGTAATCTGGAACATCGTATGGGTAGTCTATGTCCCAGTTGGCATAACC
	Reverse	TACCCATACGATGTTCCAGATTACGCTCTATTCACCTACATGCGCTTTGATGC
HA-142	Forward	AGCGTAATCTGGAACATCGTATGGGTACTCTACCTTTCTACGCATTTGCG
	Reverse	TACCCATACGATGTTCCAGATTACGCTACCGGGCAAGTTGTCCCAC
HA-149	Forward	AGCGTAATCTGGAACATCGTATGGGTAGGGTGTGCACGCAACAAAAG
	Reverse	TACCCATACGATGTTCCAGATTACGCTCAATTGCTCCAATATATGTTTGTGCCAC
MYC-157	Forward	CAGATCCTCTTCAGAGATGAGTTTCTGCTCCCTGGAGCCCCTAAGCCAG
	Reverse	AATGAGCAGAAACTCATCTCTGAAGAGGATCACAAACATATATTGGAGCAATTGTGGGAC
MYC-175	Forward	CAGATCCTCTTCAGAGATGAGTTTCTGCTCCCCTCAGTTTTTGTCAAGCTGTCAG
	Reverse	AATGAGCAGAAACTCATCTCTGAAGAGGATAGTGGCGGTTTGCCATG
MYC-183	Forward	CAGATCCTCTTCAGAGATGAGTTTCTGCTCTCAGACCCTCCAGCGCAG
	Reverse	AATGAGCAGAAACTCATCTCTGAAGAGGATCAGCTTGACAAAAACTGAGGGGTTAG
MYC-186	Forward	CAGATCCTCTTCAGAGATGAGTTTCTGCTCCCAGCGCAGGTTTCAGTG
	Reverse	AATGAGCAGAAACTCATCTCTGAAGAGGATAGGGTCTGACAGCTTGACAAAAAC
MYC-239	Forward	CAGATCCTCTTCAGAGATGAGTTTCTGCTCACCTCCAAGTCTAAGTACCCTTTAGTG
	Reverse	AATGAGCAGAAACTCATCTCTGAAGAGGATCCCCACAGTCCGCACTG
MYC-244	Forward	CAGATCCTCTTCAGAGATGAGTTTCTGCTCCCTTTAGTGGTTAGGATTTACATGAGAATGAAGC
	Reverse	AATGAGCAGAAACTCATCTCTGAAGAGGATGTACTTAGACTTGGAGGTCCCCACAG
HA-157	Forward	AGCGTAATCTGGAACATCGTATGGGTACCTGGAGCCCCTAAGCCAG
	Reverse	TACCCATACGATGTTCCAGATTACGCTCACAAACATATATTGGAGCAATTGTGGGAC
HA-175	Forward	AGCGTAATCTGGAACATCGTATGGGTACCCTCAGTTTTTGTCAAGCTGTCAG
	Reverse	TACCCATACGATGTTCCAGATTACGCTAGTGGCGGTTTGCCATG
HA -183	Forward	AGCGTAATCTGGAACATCGTATGGGTATCAGACCCTCCAGCGCAG
	Reverse	TACCCATACGATGTTCCAGATTACGCTCAGCTTGACAAAAACTGAGGGGTTAG
HA-186	Forward	AGCGTAATCTGGAACATCGTATGGGTACCAGCGCAGGTTTCAGTG
	Reverse	TACCCATACGATGTTCCAGATTACGCTAGGGTCTGACAGCTTGACAAAAAC
HA-239	Forward	AGCGTAATCTGGAACATCGTATGGGTAACCTCCAAGTCTAAGTACCCTTTAGTG
	Reverse	TACCCATACGATGTTCCAGATTACGCTCCCCACAGTCCGCACTG
HA-244	Forward	AGCGTAATCTGGAACATCGTATGGGTACCTTTAGTGGTTAGGATTTACATGAGAATGAAGC
	Reverse	TACCCATACGATGTTCCAGATTACGCTGTACTTAGACTTGGAGGTCCCCACAG
FLAG -97	Forward	GATTACAAGGATGACGACGATAAGGAGGGCACAACTAACCCAAATG
	Reverse	CGTCATCCTTGTAATCAAGAGGGAGATCTATCTCTCCAACT
FLAG -98	Forward	GATTACAAGGATGACGACGATAAGGGCACAACTAACCCAAATGGTTATGCC
	Reverse	CGTCATCCTTGTAATCCTCAAGAGGGAGATCTATCTCTCCAAC
FLAG -99	Forward	GATTACAAGGATGACGACGATAAGACAACTAACCCAAATGGTTATGCC
	Reverse	CGTCATCCTTGTAATCGCCCTCAAGAGGGAGATCTATC
FLAG-100	Forward	GATTACAAGGATGACGACGATAAGACTAACCCAAATGGTTATGCCAAC
	Reverse	CGTCATCCTTGTAATCTGTGCCCTCAAGAGGGAGAT
FLAG-101	Forward	GATTACAAGGATGACGACGATAAGAACCCAAATGGTTATGCCAACT
	Reverse	CGTCATCCTTGTAATCAGTTGTGCCCTCAAGAGGG
FLAG-102	Forward	GATTACAAGGATGACGACGATAAGCCAAATGGTTATGCCAACTGGG
	Reverse	CGTCATCCTTGTAATCGTTAGTTGTGCCCTCAAGAGGG

FLAG-103	Forward	GATTACAAGGATGACGACGATAAGAATGGTTATGCCAACTGGGACAT
	Reverse	CGTCATCCTTGTAATCTGGGTTAGTTGTGCCCTCAAG
FLAG-104	Forward	GATTACAAGGATGACGACGATAAGGGTTATGCCAACTGGGACAT
	Reverse	CGTCATCCTTGTAATCATTTGGGTTAGTTGTGCCCTC
HA-97	Forward	CATACGATGTTCCAGATTACGCTGAGGGCACAACTAACCCAAATG
	Reverse	GTAATCTGGAACATCGTATGGGTAAAGAGGGAGATCTATCTCTCCAACT
HA-98	Forward	CATACGATGTTCCAGATTACGCTGGCACAACTAACCCAAATGGT
	Reverse	GTAATCTGGAACATCGTATGGGTACTCAAGAGGGAGATCTATCTCTCCAAC
HA-99	Forward	CATACGATGTTCCAGATTACGCTACAACTAACCCAAATGGTTATGCC
	Reverse	GTAATCTGGAACATCGTATGGGTAGCCCTCAAGAGGGAGATCTATC
HA-100	Forward	CATACGATGTTCCAGATTACGCTACTAACCCAAATGGTTATGCCAAC
	Reverse	GTAATCTGGAACATCGTATGGGTATGTGCCCTCAAGAGGGAGAT
HA-101	Forward	CATACGATGTTCCAGATTACGCTAACCCAAATGGTTATGCCAACTG
	Reverse	GTAATCTGGAACATCGTATGGGTAAGTTGTGCCCTCAAGAGGG
HA-102	Forward	CATACGATGTTCCAGATTACGCTCCAAATGGTTATGCCAACTGGG
	Reverse	GTAATCTGGAACATCGTATGGGTAGTTAGTTGTGCCCTCAAGAGGG
HA-103	Forward	CATACGATGTTCCAGATTACGCTAATGGTTATGCCAACTGGGACAT
	Reverse	GTAATCTGGAACATCGTATGGGTATGGGTTAGTTGTGCCCTCAAG
HA-104	Forward	CATACGATGTTCCAGATTACGCTGGTTATGCCAACTGGGACATAG
	Reverse	GTAATCTGGAACATCGTATGGGTAATTTGGGTTAGTTGTGCCCTC
MYC-97	Forward	GAAACTCATCTCTGAAGAGGATCTGGAGGGCACAACTAACCCAAATG
	Reverse	CTTCAGAGATGAGTTTCTGCTCAAGAGGGAGATCTATCTCTCCAACT
MYC-98	Forward	GAAACTCATCTCTGAAGAGGATCTGGGCACAACTAACCCAAATGGT
	Reverse	CTTCAGAGATGAGTTTCTGCTCCTCAAGAGGGAGATCTATCTCTCCAAC
MYC-99	Forward	GAAACTCATCTCTGAAGAGGATCTGACAACTAACCCAAATGGTTATGCC
	Reverse	CTTCAGAGATGAGTTTCTGCTCGCCCTCAAGAGGGAGATCTATC
MYC-100	Forward	GAAACTCATCTCTGAAGAGGATCTGACTAACCCAAATGGTTATGCCAAC
	Reverse	CTTCAGAGATGAGTTTCTGCTCTGTGCCCTCAAGAGGGAGAT
MYC-101	Forward	GAAACTCATCTCTGAAGAGGATCTGAACCCAAATGGTTATGCCAACT
	Reverse	CTTCAGAGATGAGTTTCTGCTCAGTTGTGCCCTCAAGAGGG
MYC-102	Forward	GAAACTCATCTCTGAAGAGGATCTGCCAAATGGTTATGCCAACTGGG
	Reverse	CTTCAGAGATGAGTTTCTGCTCGTTAGTTGTGCCCTCAAGAGGG
MYC-103	Forward	GAAACTCATCTCTGAAGAGGATCTGAATGGTTATGCCAACTGGGACAT
	Reverse	CTTCAGAGATGAGTTTCTGCTCTGGGTTAGTTGTGCCCTCAAG
MYC-104	Forward	GAAACTCATCTCTGAAGAGGATCTGGGTTATGCCAACTGGGACAT
	Reverse	CTTCAGAGATGAGTTTCTGCTCATTTGGGTTAGTTGTGCCCTC
CBP-100	Forward	GCCGCCAACAGGTTCAAGAAGATCAGCAGCAGCGGCGCCCTGACTAACCCAAAT GGTTATGCCAACTG
	Reverse	CTTCTTGAACCTGTTGGCGGCGCTCACGGCGATGAAGTTCTTCTTCCACCTTCTCTTT GTGCCCTCAAGAGGGAGAT
VSV-100	Forward	TATACAGACATAGAGATGAACCGACTTGGAAAGACTAACCCAAATGGTTATGCCA ACTG
	Reverse	CTTTCCAAGTCGGTTCATCTCTATGTCTGTATATGTGCCCTCAAGAGGGAGATC
V5-100	Forward	CTATCCCTAACCCTCTCCTCGGTCTCGATTCTACGACTAACCCAAATGGTTATGCCA ACTG
	Reverse	CGAGGAGAGGGTTAGGGATAGGCTTACCTGTGCCCTCAAGAGGGAGATC
S-100	Forward	CTGCTAAATTCGAACGCCAGCACATCGACAGCACTAACCCAAATGGTTATGCCAA CTG
	Reverse	CTGGCGTTCGAATTTAGCAGCAGCGGTTTCTTTTGTGCCCTCAAGAGGGAGATC
OPSIN-100	Forward	CTACGTGCCTTTCTCCAACAAGACGGGCACTAACCCAAATGGTTATGCCAACTG
	Reverse	GTTGGAGAAAGGCACGTAGAAGTTTGGGCCTGTGCCCTCAAGAGGGAGATC

HSV-100	Forward	CAGCCAGAACTCGCCCCGGAAGACCCCGAGGATACTAACCCAAATGGTTATGCCAACTG
	Reverse	ATCCTCGGGGTCTTCCGGGGCGAGTTCTGGCTGTGTGCCCTCAAGAGGGAGATC
ALFA-100	Forward	GAAGAACTCAGACGGCGCTTGACGGAGACTAACCCAAATGGTTATGCCAACTG
	Reverse	CAAGCGCCGTCTGAGTTCTTCCTCCAGCCGTGATGTGCCCTCAAGAGGGAGATC
AcV5-100	Forward	TCTTGGAAAGATGCGAGCGGCTGGTCTACTAACCCAAATGGTTATGCCAACTG
	Reverse	AGACCAGCCGCTCGCATCTTTCCAAGATGTGCCCTCAAGAGGGAGATC
GLU-GLU-100	Forward	GAGTACATGCCAATGGAGACTAACCCAAATGGTTATGCCAACTG
	Reverse	CTCCATTGGCATGTACTCTGTGCCCTCAAGAGGGAGATC

Table S2. PCR primer sequences for construction of Recombinant EV-A71 Infectious Clone.

Name	sequences (5’→3’)
Forward-rEV-A71-VP1-S	CTGCTAAATTCGAACGCCAGCACATCGACAGCACTAACCCAAATGGTTATGCCAACTG
Reverse-rEV-A71-VP1-S	CTGGCGTTCGAATTTAGCAGCAGCGGTTTCTTTTGTGCCCTCAAGAGGGAGATC
Forward-rEV-A71-VP1-V5	CTATCCCTAACCCTCTCCTCGGTCTCGATTCTACGACTAACCCAAATGGTTATGCCAACTG
Reverse-rEV-A71-VP1-V5	CGAGGAGAGGGTTAGGGATAGGCTTACCTGTGCCCTCAAGAGGGAGATC

Table S3. PCR primer sequences for detection of Recombinant Virus Tags.

Name	sequences (5’→3’)
Forward-rEV-A71-VP1	GACTACTACACCACAGGGTTAGT
Reverse-rEV-A71-VP1	AAGAGTGGTGATCGCTGTGC

Table S4. qPCR primer sequences.

Name	sequences (5’→3’)
Forward-rEV-A71-VP1-DL	GGAGATAGCGTGAGCAGAGC
Reverse-rEV-A71-VP1-DL	TCTCCAACTAATCCCGCCCT

Reference

1. Lyu, K.; Wang, G.C.; He, Y.L.; Han, J.F.; Ye, Q.; Qin, C.F.; Chen, R. Crystal structures of enterovirus 71 (EV71) recombinant virus particles provide insights into vaccine design. *J. Biol. Chem.* **2015**, *290*, 3198–3208, doi:10.1074/jbc.M114.624536.
